# Optimization of Microbial Consortium Formulation for Oily Food Waste Composting Using Mixture Design Methodology

**DOI:** 10.3390/microorganisms13092066

**Published:** 2025-09-05

**Authors:** Yun Zhang, Yujun Shen, Jingtao Ding, Haibin Zhou, Hang Zhao, Hongsheng Cheng, Pengxiang Xu, Yiwei Qin, Yang Jia

**Affiliations:** 1 Institute of Energy and Environmental Protection, Academy of Agricultural Planning & Engineering, Ministry of Agriculture and Rural Affairs, Beijing 100125, China; yuncutie@163.com (Y.Z.); dingjingtao@163.com (J.D.); nxzhb66@163.com (H.Z.); zhaohang08@outlook.com (H.Z.); steerfeng@163.com (H.C.); xpx527@126.com (P.X.); qinyw6615@163.com (Y.Q.); a451357808@163.com (Y.J.); 2 Key Laboratory of Technologies and Models for Cyclic Utilization from Agricultural Resources, Ministry of Agriculture and Rural Affairs, Beijing 100125, China

**Keywords:** compound microbial agents, simplex-lattice mixture design, high oil-degrading, composting, rural food waste

## Abstract

The efficient compounding of microbial agents for use in aerobic composting processes is a pressing problem that needs to be addressed. This work focused on the lack of effective oil-degrading microorganisms and the challenges in formulating microbial consortia during the composting of food waste (FW). Following the isolation of three bacteria and three fungi with high oil-degrading ability, a simplex-lattice mixture design methodology was used to conduct compounding within and between groups of bacteria and fungi. Three special cubic response models were successfully developed and validated by performing an analysis of variance. From our analysis, it was demonstrated that the three models had high R^2^ values of 96.06%, 97.18%, and 96.27%. The global solution of the mixture optimization predicted the optimal value for a blend comprising 11.83% *Agrobacterium tumefaciens*, 8.10% *Pseudomonas geniculata*, 10.97% *Luteibacter rhizovicinus*, 20.9% *Simplicillium cylindrosporum*, 22.3% *Fusarium proliferatum*, and 25.9% *Simplicillium lanosoniveum.* Thus, these proportions were considered the optimal combination of strains for oil degradation during FW composting. Composting verification in a 60 L fermenter revealed that the composite microbial agent group had a 31.3% higher oil degradation efficiency than the control group. This work provides valuable insights for the compounding of microbial agents and the resource utilization of rural FW.

## 1. Introduction

Food waste (FW), including food residues and kitchen waste, is considered a significant component of municipal and rural solid waste [1]. Since the full-scale implementation of waste classification policies in China, the total amount of untreated food waste has notably risen [2]. It is projected that throughout the 14th Five-Year Plan period (2021–2025), the daily production of FW in China will amount to approximately 2 × 10^5^ tons [3]. Rural areas in China generate nearly 294 million tons of domestic waste annually, among which FW accounts for over 50% [4]. The high costs of centralized collection and treatment, in combination with the transportation of FW in remote rural areas, lead to serious resource waste. Therefore, the classification and collection of FW for local utilization has become more important than ever. Although various approaches exist for the valorization of FW, composting remains the most cost-effective method, yielding stable and carbon-rich materials that enhance soil quality [5]. Studies indicate that FW is characterized by significant organic and lipid content, which varies between 17% and 42%, along with high salt concentrations and considerable moisture levels [6,7]. Excessive oil tends to form an oil film on the pile, which inhibits oxygen from entering the FW surface. This decreases the maturation and transformation efficiency of aerobic composting, which negatively impacts the compost quality [8].

Exogenous microbial inoculants can regulate the microbial community, enhancing the aerobic fermentation efficiency of FW and improving the compost quality [9]. According to literature reports, the addition of oil-degrading microorganisms can enhance oil degradation efficiency and promote compost humification by optimizing the microbial diversity [10]. For example, Zhang et al. [11] reported on a salt-tolerant and oil-degrading *Bacillus safensis* YM1 strain, demonstrating that the application of 0.5% YM1 inoculum in compost could significantly reduce the oil content and chloride ion concentration by 19.7% and 8.1%, respectively. Additionally, Wang et al. [12] found that the introduction of *Bacillus subtilis* and *Bacillus glycinifermentans* into FW composting could significantly boost microbial populations within the first three days, resulting in a 39.96% reduction in oil content after five days. Studies have also found that, due to the complexity of the oil degradation process, it may be difficult for a single bacterial agent to achieve full-chain degradation of oil, and the application of mixed microbial agents may be more effective [13]. Jing et al. [14] and Li et al. [15] conducted orthogonal experiments for the compounding of microbial agents. However, this method could only be carried out at a limited level and obtaining the actual optimal ratio proved challenging.

Mixture design methods are valuable statistical tools used to obtain the maximum desired effect using the minimum number of experiments [16]. Among these methods, the one-factor-at-a-time (OFAT) approach is commonly used for optimizing microbial inoculant formulations and ratio selection; however, it suffers from limitations such as low efficiency and suboptimal accuracy [17]. Simplex-lattice mixture design is another mixture design method. As a statistical tool, it can model the impact of various process factors on a system response, both individually and through their cumulative interactions, providing an indication of the optimal operating region [18,19]. One key benefit is its efficiency, enabling the calculation of multiple parameters with a reduced number of experiments while still delivering precise quantitative outcomes [20]. However, it is rarely used in the field of microbial agent compounding at present.

Based on these approaches, this study systematically investigated efficient oil-degrading microbial agents for composting FW in rural areas. Based on the previous screening of six oil-degrading strains in our laboratory, a combination experiment of microbial agents was performed. A self-designed small-scale fermentation system was used to simulate the composting process. The experimental plan was formulated using the simplex-lattice mixture design methodology. First, the optimal ratios of three bacterial strains and three fungal strains were explored. On this basis, compounding between the bacterial and fungal groups was performed. The aim was to (1) improve the compounding efficiency of microbial agents, (2) develop a method for compound microbial agent formulation, (3) determine the optimal ratio of compounded microbial agents, and (4) provide new methods and ideas for the efficient development of microbially enhanced composting technology.

## 2. Materials and Methods

### 2.1. Raw Materials and Equipment

**Figure 1:**
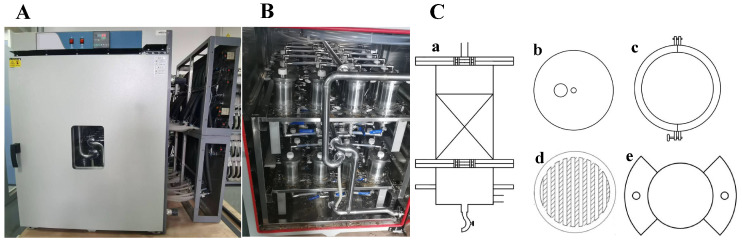
Diagram of the small-scale fermentation simulation device: (**A**) Main view of the physical object; (**B**) internal structure of the physical object; and (**C**) schematic diagram of the small-scale fermentation tank, showing (**a**) tank body, (**b**) upper cover, (**c**) sealing ring clamp, (**d**) support plate, and (**e**) fixed gasket.

Due to the large scale of natural composting piles, research on microbial agent formulation requires substantial amounts of inocula, leading to high costs and low formulation efficiency. Therefore, following the experimental approach of Wu et al. [21], a self-designed small-scale fermentation simulation system for composting and microbial formulation tests was employed (Figure 1). The system included an automatic programmable temperature-controlled chamber, an air and water supply system, and a mini-fermenter. Typically, temperature is regarded as a crucial parameter that is closely linked to composting efficiency and microbial activity [22]. The temperature-controlled chamber could be set to multiple temperature stages based on actual composting conditions and maintained each temperature for a specific duration, simulating natural temperature variations during composting. Detailed temperature settings are presented in the Supplemental Materials. Two rounds of composting simulation experiments were conducted. The first was intra-group formulation, that is, separate ratio optimization among the three bacteria or the three fungi. The second was inter-group formulation, that is, ratio optimization between bacteria and fungi based on the best intra-group combinations.

The air and water supply system consisted of an air compressor, rotameter, bubbler-type humidifier, and time relay. The air compressor was controlled by the time relay, enabling intermittent supply of oxygen and moisture into the fermenter. The mini-fermenter comprised a tank, supporting plate, sampling port, air inlet, and leachate outlet (as shown in Figure 1C). The mixed feedstock was placed on the supporting plate, with air supplied upward from the bottom and leachate draining from the top to the bottom. The inner diameter of the fermenter was 86 mm, the packing zone height was 125 mm, and the maximum packing volume was 725 mL.

The FW was taken from the canteen of the Planning and Design Institute of the Ministry of Agriculture and Rural Affairs. Corn stalks were purchased and cut into lengths of 1–2 cm. The specific parameters of the materials used in the two processes are presented in Table 1. The material filling volume of each fermentation tank was approximately 600 mL. During the fermentation process, the ventilation rate was maintained at 0.2 L/(min·kg) dry mass, and the ventilation system was ventilated on a schedule of 30 min on and 30 min off.

### 2.2. Strain and Mixture Design

**Figure 2:**
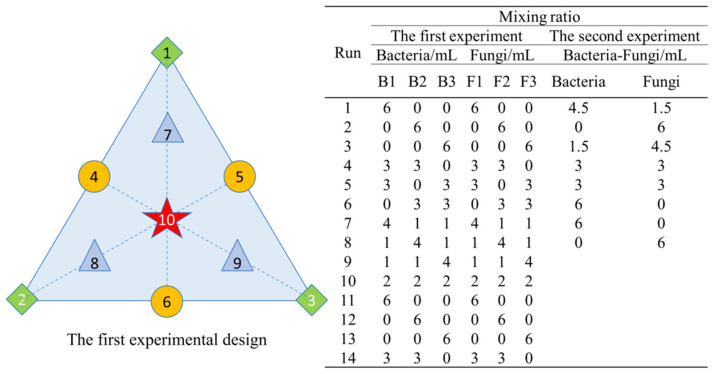
Experimental design: three-and two-component systems with schematic diagram (The numbers in the schematic diagram have the same meaning as the Run column (entries 1 to 10) in the three-component experimental of the table).

To avoid antagonism between strains, all microbial species were isolated from the same initial microbial source. A total of six oil-degrading strains were obtained, including three bacteria, *Agrobacterium tumefaciens* (B1), *Pseudomonas geniculata* (B2), and *Luteibacter rhizovicinus* (B3), and three fungi, *Simplicillium cylindrosporum* (F1), *Fusarium proliferatum* (F2), and *Simplicillium lanosoniveum* (F3). The isolation and identification process, as well as the oil-degradation capabilities of these strains are detailed in previous studies. The mixed design of the experiment was determined using Design-Expert 13 software, which involved calculating a three-dimensional (3D) response surface and performing an analysis of variance. The specific designs of the two experiments are shown in Figure 2. A CK group without microbial inoculation was included in each experiment. Based on the substrate volume, the inoculation level for both experiments was approximately 1% (*w*/*w*), equivalent to a total inoculum volume of 6 mL. The viable cell count in each microbial suspension was determined using the gradient dilution method. The suspensions were serially diluted with physiological saline, and a fixed volume of each dilution was evenly spread onto agar plates. Each dilution was performed in triplicate and incubated in a constant-temperature incubator at 30 °C. After a specific period, the number of individual colonies on each plate was recorded. The viable cell concentration was then calculated based on the average colony count and the corresponding dilution factor and recorded as CFU/mL. To improve the homogeneity of fungal suspension, mycelial clumps were dispersed by vortexing with glass beads prior to viability determination. The composition of the culture medium was as follows: beef extract (5.0 g), peptone (10.0 g) and NaCl (5.0 g). The cell concentration of all six strains was adjusted to 1.0 × 10^10^ CFU/mL according to the viable cell count. The addition levels of each component ranged from 0 to 6 mL.

### 2.3. Sample Collection

Oil degradation mostly occurs during the thermophilic and cooling phases of composting, and changes in oil composition are no longer pronounced in the maturity phase of composting [23]. By day 27, the composting process had progressed to the maturity phase, with oil degradation showing minimal further changes. Therefore, to shorten the experimental duration, the oil degradation efficiency was assessed based on samples collected on day 27. Well-mixed compost samples (50 g) were taken on days 0, 3, 7, 20, and 27 during the composting process. Three replicates were measured for each group. The physicochemical analysis samples were stored at 4 °C and analyzed using the methods described in the Supplementary Materials, as reported by Liu et al. [6].

### 2.4. Composting Validation Test

The wet weight ratio of FW to cornstalk was 4:1, with a moisture content of approximately 55~65%, a carbon-to-nitrogen ratio (C/N) of 20–30, and a pH of 4~7. The experiment was conducted in a 60 L self-developed fermenter (400 mm diameter and 450 mm height). For the convenience of pile-turning, the material filling rate was approximately 80%, resulting in an actual material volume of approximately 48 L. The group without added microbial agents was the blank control (CK). The other three groups were treated based on the simplex-lattice mixture design fitting results, and bacterial, fungal, and bacterial–fungal mixed microbial solutions were prepared as inoculants. The microbial solution activity was 2 × 10^12^ CFU/mL, and the inoculation volume for each group was 5% (*w*/*w*). During the fermentation process, the ventilation rate was maintained at 0.2 L/(min·kg) dry mass, and the ventilation system was controlled by a time relay, which ventilated on a schedule of 30 min on and 30 min off. Homogeneous composting samples (200 g) were collected from the top, middle, and bottom of the fermentation tank at 0, 3, 7, 14, 21, 28, 35, and 42 days of composting to determine the efficiency of oil degradation.

### 2.5. Analytical Methods

To verify the feasibility of the temperature control system in the fermentation simulation setup, the temperature inside the small fermenter was measured daily using a thermometer. To assess the system’s effectiveness in simulating real-world composting conditions, key physicochemical parameters of the composting material, including pH, moisture content, carbon-to-nitrogen ratio, electrical conductivity (EC), and germination index (GI), were monitored throughout the process. The temperature was measured using a thermometer (TP101, Hengshui, China). The fermented wet sample was mixed with deionized water at a 1/10 (m/v) ratio. The sample mixture was shaken at 160 rpm at 25 °C for 1 h and then centrifuged at 4500 rpm for 10 min. After filtering, the supernatant was collected to determine pH and seed germination index (GI). The pH was measured using a portable pH meter (PHBJ-260, Leici, Shanghai, China). The fresh samples (m1) were dried in an electric thermostatic oven (DHG-9030 A, Hengtaifengke, Beijng, China) at 105 °C, cooled in a dryer, and weighed (m2). The moisture content of the materials was recorded as [(m1 − m2)/m1]. Total carbon and total nitrogen contents were measured using an elemental analyzer (Flash 2000, Thermo Fisher Scientific, Waltham, MA, USA). Detailed results are provided in the Supplementary Material.

Since the purpose of this experiment was to obtain microbial agents with a better oil degradation effect, the oil degradation efficiency was taken as the output response value of the mixture design to fit the compounding results of the microbial agents. The oil content was determined by Soxhlet extraction (GB/T 5009.6-2016, Chinese Standard) [24]. The total nitrogen (TN) concentration of FW compost was quantified using Kjeldahl’s method (NY/T 525-2021) [25], and the total phosphorus (TP) and total potassium (TK) concentrations were quantified using inductively coupled plasma spectroscopy (NY/T525-2021).

The first stage of the statistical analysis involved ANOVA, and the results obtained were analyzed using linear, quadratic, and special cubic models. The *p*-value and the coefficient of determination adjusted R^2^ (Adj. R^2^) were used as the acceptance criteria for the model.

### 2.6. Data Analysis

The mixture design data were calculated using Design-Expert 13.0.1.0 software, and the accuracy of the model was verified through 3D response surface and variance analysis. All data were analyzed and plotted using WPS Office, Origin 8.5.

## 3. Results

### 3.1. Oil Degradation Efficiency in Three Groups of Experiments

After 27 days of composting, the oil degradation efficiency of each experimental group was determined (Table 2). The higher oil degradation in groups inoculated with higher-efficiency oil-degrading strains compared to the CK groups (5.61% and 4.85%) indicated that inoculation with oil-degrading strains improved oil degradation during composting. Overall, the oil degradation efficiency of the fungal group was better than that of the bacterial group, indicating that several fungal strains were more effective during the composting process. Among the three groups, the bacterial–fungal complex group was the worst. This may have been due to differences in the basic physical and chemical properties of the materials in the two experiments. The oil content of the initial materials in the first composting was 11.93%, and that in the second composting was 27.87%. However, the average oil degradation amounts in the bacterial, fungal and bacterial–fungal groups were 2.27%, 3.14% and 3.67%, respectively, with the bacterial–fungal group exhibiting the highest oil degradation amount.

Additionally, the trends in basic physicochemical parameters of the fermentation materials in each tank, such as temperature, pH, moisture content, C/N ratio, and GI confirmed that the small-scale simulation fermenter could ensure the smooth progression of the fermentation process (see Supplementary Materials). Therefore, this simulation system was suitable as a fermentation device for testing microbial agent formulations.

### 3.2. Analysis of Variance and Model Calibration

A higher Adj. R^2^ value, approaching 1, indicates a better model fit [26]. The summary of the fitting of different models indicated that special cubic models were applicable to bacterial, fungal, and bacterial–fungal groups (Table 3). The *p*-values of the bacterial, fungal and bacterial–fungal special cubic models were all < 0.05, indicating that these models were statistically valid for predicting the response variables. Additional details can be found in Supplementary Materials. The model F-values for the bacterial, fungal, and bacterial–fungal groups were 28.45, 40.25, and 34.39, respectively, demonstrating that all three models were highly significant at the 95% confidence level or above. Additionally, there was only a 0.01%, <0.01%, and 0.26% chance that the large F-values for the bacterial, fungal, and bacterial–fungal groups, respectively, could occur due to noise. The R^2^ values for the bacterial, fungal, and bacterial–fungal models were 96.06%, 97.18%, and 96.27%, respectively, indicating that all three models fit the experimental data well. However, it is important to note that adding more variables to a model can artificially inflate the R^2^ value, even if the additional variables are not statistically significant. To prevent overestimation, the Adj. R^2^ was used as a more reliable statistical measure. The Adj. R^2^ and predicted R^2^ (Pred. R^2^) values were 92.68% and 82.58% for the bacterial model, 94.77% and 87.56% for the fungal model, and 93.47% and 82.03% for the bacterial–fungal model, respectively, indicating that all fitted models exhibited a high level of statistical significance.

Adequate precision (Adeq. precision) is a metric that evaluates the signal-to-noise ratio by comparing the range of predicted values at the design points to the average prediction error. A ratio greater than 4 is desirable, and the ratio of all three models indicates an adequate signal. These models can be used to navigate the design space.

### 3.3. Model Adequacy Test

**Figure 3:**
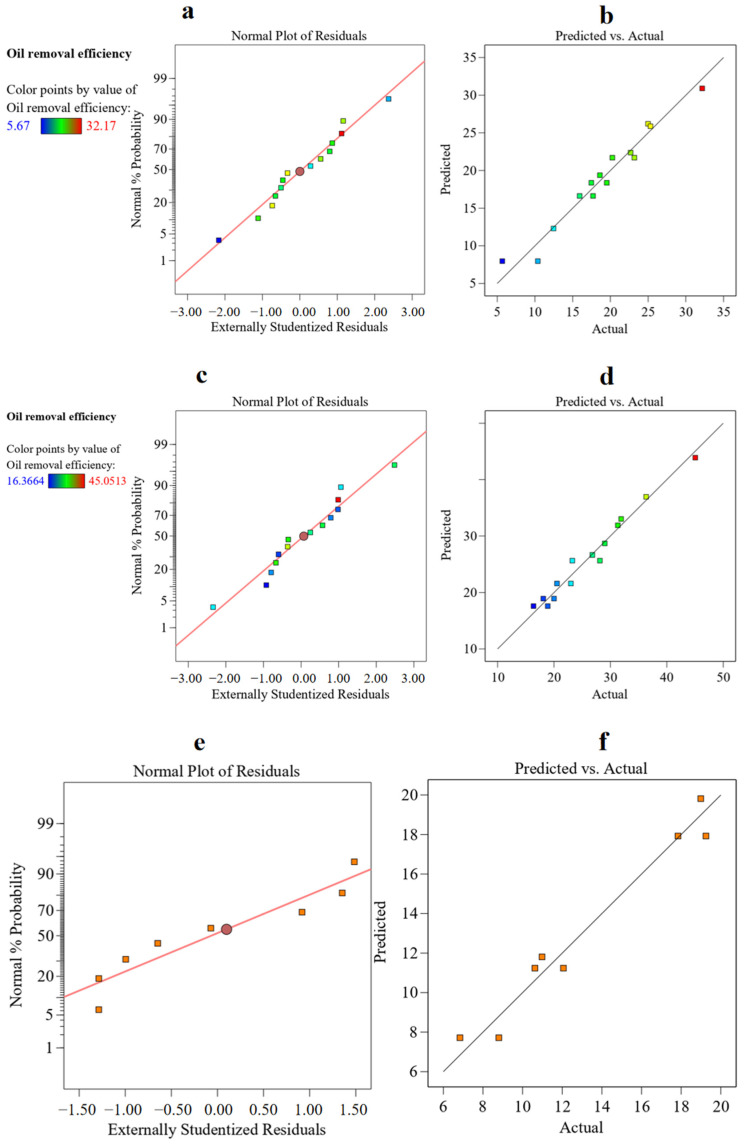
Diagrams showing the relationship between predicted and experimental values for the (**a**) bacterial group, (**c**) fungal group, and (**e**) bacterial–fungal group. Normal probability plots of the residual errors for the (**b**) bacterial group (**d**), fungal group, and (**f**) bacterial–fungal group (the square represented experimental values).

The practical applicability of the mixed-strain model (Table 4) in composting systems was assessed by both graphical and numerical approaches [27]. Graphical methods were used to examine the residuals of the model, which represent the differences between the observed and predicted values, while numerical approaches focused on determining the R^2^ and Adj. R^2^ values. In most cases, it is essential to validate the fitted model to ensure that it accurately reflects the real-world scenario. To assess the normality of residuals, a normal probability plot was used, as shown in Figure 3a,c,e, under the assumption that the residuals followed a typical distribution. As the data points were closely aligned with the straight reference line, no response transformation was necessary, and there were no apparent violations of the normality assumption in the regression model equations.

Figure 3b–f shows that the data points in the plots were closely aligned with a straight line, indicating a strong correlation. The R^2^ values between the predicted and the actual response values were 96.06%, 97.18%, and 96.27% for the bacterial, fungal, and bacterial–fungal models, demonstrating that the regression models could effectively capture the trends in the experimental data. Additionally, the developed models illustrated that the difference between Adj. R^2^ and Pred. R^2^ was less than 20%, indicating that the developed models were suitable for predicting oil removal efficiency in the different mixed-strain inoculum compost.

### 3.4. Effects of Different Strains in the Bacterial, Fungal, and Bacterial–Fungal Groups

The influence of the impact factor on the fitting model was evaluated using a method that combines numerical and graphical approaches. According to the model fitting results, a negative partial regression coefficient in the non-linear term indicates an antagonistic interaction among components in a binary or ternary mixture, whereas a positive coefficient suggests a synergistic effect between the components [28].

**Figure 4:**
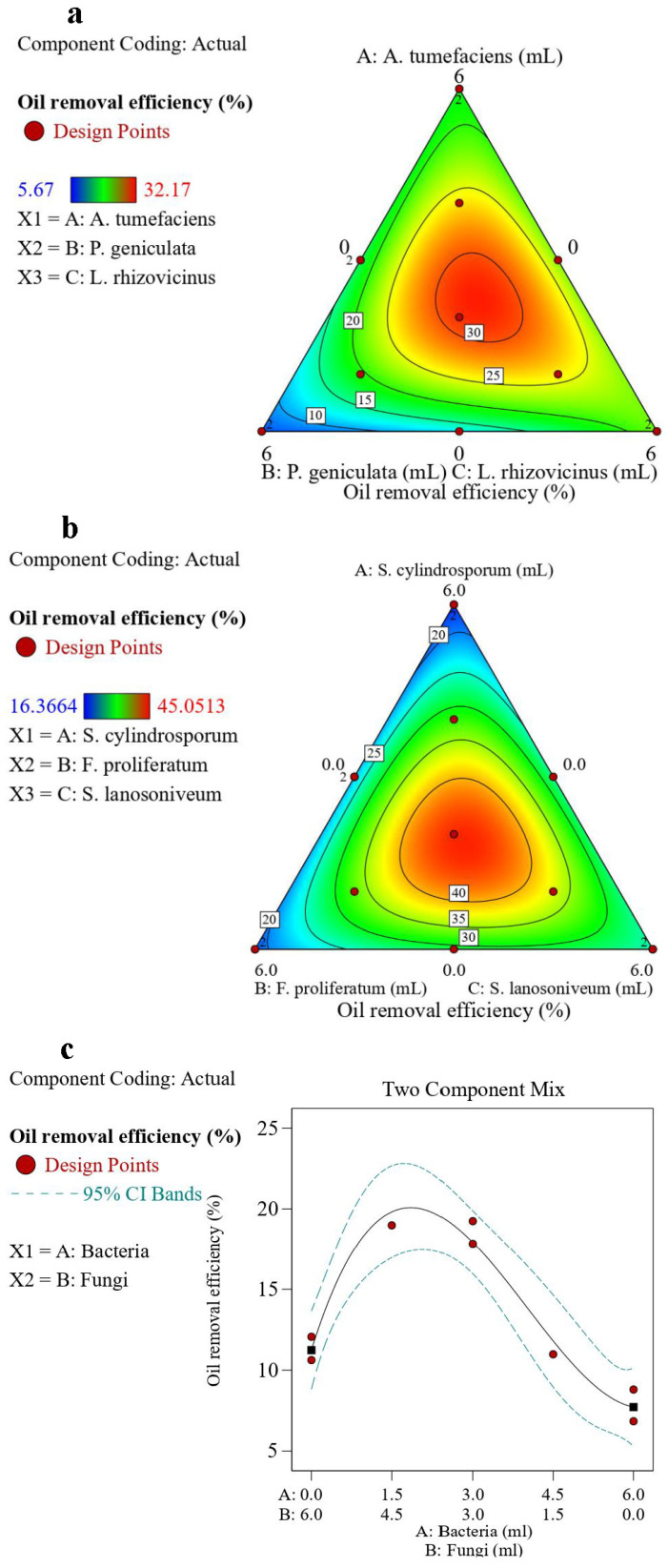
Oil removal efficiency contours under varying proportions of the (**a**) bacterial group, (**b**) fungal group, and (**c**) bacterial–fungal group (the squares represented the boundary value).

Figure 4 and Table 4 show that for the bacterial model, the presence of the three bacteria positively affected oil degradation. However, there was an antagonistic effect between bacteria B and C. Compared with B, A and C had more beneficial effects on improving the oil removal efficiency. All fungi had a positive effect on oil degradation, and the effect among the three fungi was synergistic. This indicated that the fungi had a better overall oil-degrading efficiency compared to the bacteria. As shown in Figure 4c, the peak was closer to the direction of the fungi, indicating that the fungal group had better oil-degrading performance than the bacterial group. This was also reflected in the negative coefficient of the last term of the polynomial. Compared with A and B, C had a better promoting effect on improving the oil-degrading efficiency. Bacteria and fungi also had a synergistic effect. This result indicated that the combination of the six strains for the preparing the microbial agent was reasonable.

### 3.5. Optimization of Mixture Proportions

In this study, the expected value of 1 was used to define the best mixture proportions. The optimization was extra-interactive and considered the interactions among the different independent factors and the response. The developed optimization objective was the oil removal efficiency. Equal weight values were allocated to all the optimized items.

**Figure 5:**
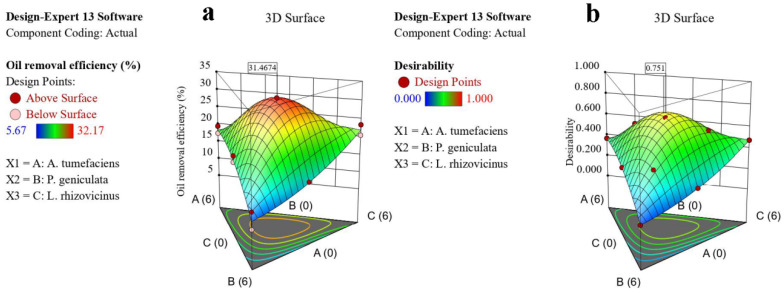

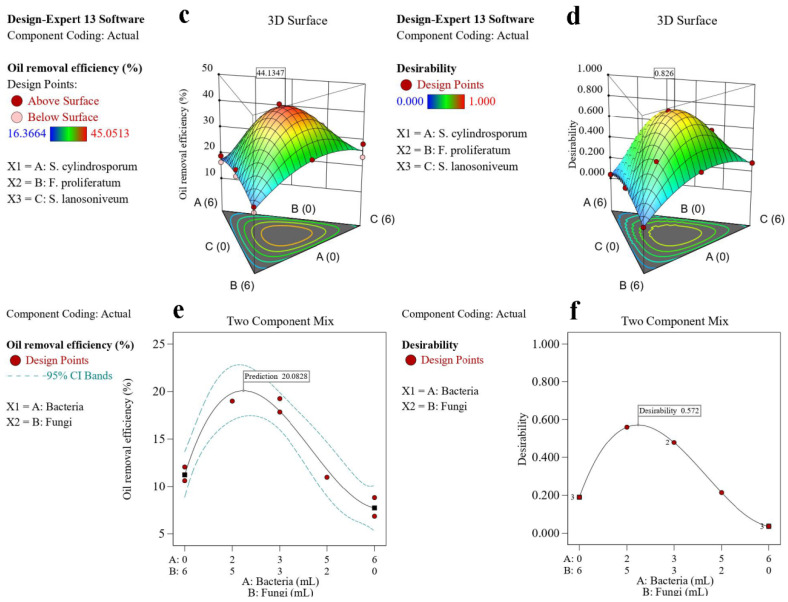
Multi-objective optimization of oil removal efficiency and desirability for the (**a**,**b**) bacterial group, (**c**,**d**) fungal group, and (**e**,**f**) bacterial–fungal group (the squares represented the boundary value).

As shown in Figure 5, the global solution for the mixture optimization in the bacterial group predicted a maximum oil removal efficiency of 31.47% for a blend of 2.30 mL *A. tumefaciens*, 1.57 mL *P. geniculata*, and 2.13 mL *L. rhizovicinus*, with a composite desirability of 0.751. In the fungal group, the mixture optimization predicted a maximum oil removal efficiency of 44.13% for a blend of 1.82 mL *S. cylindrosporum*, 1.93 mL *F. proliferatum*, and 2.25 mL *S. lanosoniveum*, with a composite desirability of 0.826. The second experiment was performed by preparing mixed solutions of bacteria and fungi based on the optimized results of the bacterial and fungal groups. In the bacterial–fungal group, the mixture optimization predicted a maximum oil removal efficiency of 20.08% for a blend of 1.85 mL bacterial mixture solution and 4.15 mL fungal mixture solution, with a composite desirability of 0.572. This was identified as the best combination of strains. After performing the calculations, the mixture optimization of the six strains predicted the optimal value for a blend comprising 11.83% *A. tumefaciens*, 8.10% *P. geniculata*, 10.97% *L. rhizovicinus*, 20.9% *S. cylindrosporum*, 22.3% *F. proliferatum*, and 25.9% *S. lanosoniveum.*

Notably, due to differences in the initial material conditions, the optimal value fitted by the model was lower than those of the bacterial and fungal groups. However, this did not affect the fact that the oil-degrading ability of the mixed bacterial–fungal group was superior to that of the individual bacterial and fungal groups. Furthermore, for this model, the magnitude of the desirability value depended on the target value of the oil removal efficiency set; therefore, it can only be used as a reference.

## 4. Discussion

### 4.1. Experimental Result Accuracy

The oil degradation performance of the six strains was reported in our previous study. In shake-flask experiments, all strains achieved oil removal efficiencies above 87% after 6 days of cultivation. The oil degradation efficiency of fungi was slightly higher than that of bacteria, which was consistent with the results from the small-scale fermentation trials in this study. This consistency further validated the feasibility of using bench-scale fermenters for the formulation of microbial consortia.

A higher oil content increases the difficulty of degradation [6]. The higher oil removal achieved in the second experiment indicated that the combination of bacteria and fungi could enhance the oil degradation ability of the microorganisms, which was consistent with the conclusion of some scholars that a combination of microbes has a better effect [29]. The coefficient of variation (C.V.%) was also used to assess the ratio of the estimated standard deviation to the mean of the observed response, serving as an indicator of the model’s reproducibility and repeatability [30]. The calculated C.V.% values were 9.58%, 3.48%, and 9.40% for the bacterial, fungal, and bacterial–fungal models, respectively. Thus, all models exhibited acceptable reproducibility, as these values were below 10% (Supplementary Materials). These results further confirmed the effectiveness of combining multiple oil-degrading strains in the formulation.

The composting verification test showed that, under the same initial conditions, with an oil content of 20%, the oil removal efficiencies of the CK group, bacterial group, fungal group, and bacterial–fungal group were 44.35, 56.8%, 66.5%, and 75.6%, respectively. Thus, the performance of the bacterial–fungal group was superior, with an increase in oil degradation efficiency of 31.3%. Thus, it could be argued that it is feasible to design experiments using the simplex-lattice mixture design methodology and perform strain compounding experiments in small-scale simulated fermenters. This method was suitable for the compounding of microbial agents.

### 4.2. Assessment of Experimental Design Completeness

For the past 50 years, microorganisms have proven to be effective in environmental protection, agricultural biotechnology, and the improved treatment of agricultural and municipal waste [31]. Current research includes commercially available microbial agents that are self-screened and purchased, while products developed from self-cultivated microbial agents are relatively limited [10,23]. Composting is performed on large piles of organic waste, so studying the formulation of microbial agents at the pile level not only demands a substantial amount of agents but also involves multiple experimental setups for single-factor and orthogonal experiments [13]. This consumes considerable human and material resources, with results that are often limited to specific experimental levels [17]. To address this issue, the size of the compost pile needs to be optimized, and faster, more accurate experimental design methods must be designed. Some studies have utilized incubators and modified culture bottles to construct composting systems for investigating the humification process, with the results demonstrating the feasibility of this approach, thereby simplifying experimental procedures [21]. The small-scale simulated fermentation device used in this study was specifically designed for composting experiments. The results showed that this device could effectively simulate the reaction conditions of a compost pile, promoting normal composting processes in small piles and creating favorable conditions for the formulation of microbial agent mixtures.

The simplex-lattice mixture design technique allows the analysis of interactions among multiple variables across varying ranges through three-dimensional visualization. It has been widely applied, including in optimizing the proportions of food manufacturers [32], extractants [33], proppants [34], and enzymatic preparations [35], as well as in the field of analytical chemistry [36]. The results of this study demonstrate that the simplex-lattice mixture design method can be effectively applied to the formulation of microbial agents. Additionally, a more accurate formulation ratio of microbial agents was achieved in this work, in contrast to the previously reported composting inoculants that were typically optimized and applied at fixed proportions, such as 1:1 or 1:1:1 [17,37]. These results break through the limitations of microbial agent configurations at specific levels, holding significant implications for the future mass production and application of microbial agents across various fields. Additionally, this method is not only suitable for exploring the optimal ratios of binary or ternary components but can also be extended to the formulation of four-component or even more complex mixtures, yielding highly accurate results. However, it should be noted that while scientific research results were precise, achieving such precision (e.g., to two decimal places) in the formulation of microbial inoculants was difficult in practice. Therefore, the approach and methodology provided in this study were feasible for calculating inoculant ratios, but in practical applications, preparing formulations near the calculated ratio will achieve similar effects, as the response surface in Figure 5 showed a peak over a certain range.

### 4.3. Application Prospects of Food Waste Composting

FW is considered a valuable source for agricultural applications, providing organic matter and nutrients to arable soils [38]. The analysis of compost characteristics from FW in this study indicated that after 42 days of composting, the groups inoculated with microorganisms had an organic matter content exceeding 80%, a C/N ratio above 12, and total N, P, and K contents of 30.23 ± 0.85 mg/g, 7.26 ± 0.07 mg/g, and 7.11 ± 0.41 mg/g, respectively. The electrical conductivity (EC) was less than 4, and the pH was approximately 7.5. Compared to literature values, this compost had higher organic matter, total N, and total P contents [39]. Zhang et al. [38] applied FW compost as a fertilizer in tobacco cultivation, demonstrating that it exerted beneficial effects by regulating soil bacterial communities, primarily through modifications in soil chemistry, which in turn indirectly enhanced soil enzyme activity and increased tobacco yield. Biochemical pathway analysis of microbial oil utilization during composting indicates that oils degraded by microorganisms became a source of energy or supplied precursors for humus. Additionally, microorganisms can synergistically degrade both oils and lignocellulose, generating more precursors and promoting the formation of humus and humic acid [6]. Through these mechanisms, oil-degrading strains not only enhance oil degradation but also promote the humification of food waste, which has positive implications for improving compost quality and, consequently, soil properties. Therefore, given China’s substantial generation of FW and extensive arable land, the application of FW compost has significant potential and economic value [40]. However, further research is needed to explore the effects of compost derived from exogenous microbial-treated FW on crops and soil, addressing issues and solutions related to its application. While the microbial degradation process of oils has been well-documented, understanding how the microbial consortia obtained in this study synergistically degrade oils and which specific stages are critical remains to be elucidated. Additionally, the risks associated with practical applications must be further assessed.

In summary, this study provides a comprehensive design methodology and conceptual framework for the development and utilization of composting microbial agents. It provides a valuable reference for addressing challenges in microbial agent formulation and holds significance for advancing formulation technologies. Furthermore, the developed microbial agent products can help address the issue of high oil content in rural FW.

## 5. Conclusions

Based on the oil removal efficiency of FW compost, a mixture design was used to optimize the compounding of microbial agents, including bacteria, fungi, and bacteria–fungi. After 14 sets for 2 and 8 sets for 1 experimental comparisons and model estimations, the optimal proportions were determined to be *A. tumefaciens*:*P. geniculata*:*L. rhizovicinus*:*S. cylindrosporum*:*F. proliferatum*:*S. lanosoniveum* = 11.83:8.10:10.97:20.9:22.3:25.9. A validation test in a 60 L composting fermenter demonstrated a 31.3% improvement in oil degradation efficiency for the group treated with the composite microbial agent compared to the untreated control group, confirming it as the optimal strain combination for oil degradation during FW composting. The approach in this study enhanced the efficiency of microbial agent compounding and provides a new avenue for optimizing the compounding of microbial agents. Additionally, this study helps solve the problem of high oil content in FW composting in rural areas of China. Furthermore, as part of the body of research exploring the potential of FW compost as a soil amendment, the microbial consortium developed in this study demonstrates significant economic value in oil degradation and soil fertility restoration.

## Figures and Tables

**Table 1:** Initial material parameters of the two simulated composting experiments.

Microbial Agent Compounding Experiment	Material	pH	Electrical Conductivity (mS/cm)	Moisture Content (%)	Total Carbon (%) ^a^	Total Nitrogen (%) ^a^	C/N ^a^
First experiment	Food waste	6.14 ± 0.04	2.64 ± 0.02	70.55 ± 0.38	46.30 ± 0.23	2.00 ± 0.06	23.17 ± 0.63
	Cornstalk	7.15 ± 0.03	3.30 ± 0.04	6.90 ± 0.14	38.94 ± 0.10	0.97 ± 0.03	39.11 ± 1.12
Second experiment	Food waste	6.71 ± 0.05	3.10 ± 0.01	72.57 ± 0.01	45.31 ± 0.04	1.86 ± 0.08	24.36 ± 1.10
	Cornstalk	7.33 ± 0.02	3.21 ± 0.00	6.44 ± 0.01	39.56 ± 0.51	1.02 ± 0.04	38.78 ± 0.97

^a^ Based on dry weight.

**Table 2:** Oil removal efficiency of the two experiments.

Run	Bacterial Group	Fungal Group	Bacterial–Fungal Group
	Oil Removal Efficiency/%		
1	17.47	18.89	10.98
2	10.38	18.10	12.06
3	20.26	28.14	18.99
4	15.92	23.00	17.84
5	22.68	26.80	19.25
6	12.46	29.00	8.80
7	24.99	31.32	6.84
8	18.61	31.91	10.62
9	25.31	36.34	
10	32.17	45.05	
11	19.52	16.37	
12	5.67	20.00	
13	23.18	23.25	
14	17.70	20.53	

**Table 3:** Fitting summary of model types.

	Source	Sequential *p*-Value	Lack of Fit *p*-Value	Adjusted R^2^	Predicted R^2^	Estimate
Bacteria	Linear	0.0329	0.0256	0.3648	0.2130	
	Quadratic	0.1394	0.0366	0.5438	–0.5138	
	**Special Cubic**	**0.0003**	**0.8278**	**0.9268**	**0.8258**	**Suggested**
Fungi	Linear	0.3940	0.0061	0.0023	–0.2922	
	Quadratic	0.0289	0.0195	0.5300	–0.6110	
	**Special Cubic**	**<0.0001**	**0.8703**	**0.9477**	**0.8756**	**Suggested**
Bacteria–Fungi	Linear	0.3181	0.0082	0.0258	–0.4730	
	Quadratic	0.0044	0.0690	0.7999	0.6930	
	**Special Cubic**	**0.0282**	**0.2884**	**0.9347**	**0.8203**	**Suggested**

**Table 4:** Polynomial equations for the compounding experiments for the three groups of microbial agents.

Compounding Group	Polynomial Models	R^2^	Adj. R^2^	Pred. R^2^
Bacteria	18.36 × A + 7.97 × B + 21.70 × C + 13.82 × AB + 9.36 × AC − 10.13 × BC + 362.87	0.9606	0.9268	0.8258
Fungi	17.6 × A + 18.93 × B + 25.66 × C + 13.38 × AB + 20.11 × AC + 25.56 × BC + 448.44 × ABC	0.9718	0.9477	0.8756
Bacteria–Fungi	7.72 × A + 11.24 × B + 33.79 × AB − 33.33 × AB (A − B)	0.9627	0.9347	0.8203

## Data Availability

The data presented in this study are available on request from the corresponding author due to privacy restrictions.

## Supplementary Materials

The following supporting information can be downloaded at: https://www.mdpi.com/article/10.3390/microorganisms13092066/s1. Table S1: Programmed temperature rise settings. Figure S1: Effects of mixture bacteria on GI (a), pH (b), moisture content (c), C/N (d) during composting of food waste with cornstalk in the small-scale simulation fermenter. Figure S2: Effects of mixture fungi on GI (a), pH (b), moisture content (c), C/N (d) during composting of food waste with cornstalk in the small-scale simulation fermenter. Figure S3: Effects of mixture bacteria and fungi on GI (a), pH (b), moisture content (c), C/N (d) during composting of food waste with cornstalk in the small-scale simulation fermenter. Figure S4: The temperature changes during the two simulation experiments of the small-scale simulation fermenter (a) the first experiment, and (b) the second experiment. Table S2: Analysis of variance (ANOVA) for the bacterial, fungal and bacterial-fungal group.

## References

[1] Awasthi M.K., Selvam A., Chan M.T., Wong J.W.C. (2017). Bio-degradation of oily food waste employing thermophilic bacterial strains. Bioresour. Technol..

[2] (2019). Notic of the Ministry of Housing and Urban-Rural Development and Other Departments on the Comprehensive Implementation of Municipal Waste Classification in Cities at and Above the Prefectural Level.

[3] Chang Y., Huang H., Zhao Z., Yin J., Zhang H., Zhang J. (2021). Prospect for the development of recycling treatment and high-value utilization technologies of food waste. Environ. Sanit. Eng..

[4] Tang F., Yu Z., Li Y., Chen L., Ma X. (2019). Catalytic co-pyrolysis behaviors, product characteristics and kinetics of rural solid waste and chlorella vulgaris. Bioresour. Technol..

[5] Yang J.W., Luyima D., Park S.J., Kim S.H., Oh T.K. (2021). Mixing sodium-chloride-rich food waste compost with livestock manure composts enhanced the agronomic performance of leaf Lettuce. Sustainability.

[6] Liu J., Shen Y., Ding J., Luo W., Zhou H., Cheng H., Wang H., Zhang X., Wang J., Xu P. (2023). High oil content inhibits humification in food waste composting by affecting microbial community succession and organic matter degradation. Bioresour. Technol..

[7] Zhou Y., Engler N., Nelles M. (2018). Symbiotic relationship between hydrothermal carbonization technology and anaerobic digestion for food waste in China. Bioresour. Technol..

[8] Meng Y., Li S., Yuan H., Zou D., Liu Y., Zhu B., Chufo A., Jaffar M., Li X. (2015). Evaluating biomethane production from anaerobic mono- and co-digestion of food waste and floatable oil (FO) skimmed from food waste. Bioresour. Technol..

[9] Chang Y., Zhou K., Yang T., Zhao X., Li R., Li J., Xu S., Feng Z., Ding X., Zhang L. (2023). *Bacillus licheniformis* inoculation promoted humification process for kitchen waste composting: Organic components transformation and bacterial metabolic mechanism. Environ. Res..

[10] Zhu L., Zhao Y., Yao X., Zhou M., Li W., Liu Z., Hu B. (2023). Inoculation enhances directional humification by increasing microbial interaction intensity in food waste composting. Chemosphere.

[11] Zhang X., Zhang D., Yan Y., Wang R., Chi Y., Zhang D., Zhou P., Chu S. (2024). Enhancing aerobic composting performance of high-salt oily food waste with *Bacillus safensis* YM1. Bioresour. Technol..

[12] Wang W., Zhao Z., Yang J., Lian X., Xie X., Chen H., Wang M., Zheng H. (2024). Application of oil-degrading agents consisted of thermophilic *Bacillus subtilis* and *Bacillus glycinifermentans* in food waste. Environ. Technol..

[13] Ke X., Hua X., Sun J., Zheng R., Zheng Y. (2021). Synergetic degradation of waste oil by constructed bacterial consortium for rapid in-situ reduction of kitchen waste. J. Biosci. Bioeng..

[14] Jing J., Wang T., Guo X., Huang P., Li C., Qu Y. (2024). Construction and application of petroleum-degrading bacterial agents: Community composition, lyophilization technology, and degradation mechanism. J. Environ. Chem. Eng..

[15] Li L., Ding X., Qian K., Ding Y., Yin Z. (2011). Effect of microbial consortia on the composting of pig manure. J. Anim. Vet. Adv..

[16] Pilkington J.L., Preston C., Gomes R.L. (2014). Comparison of response surface methodology (RSM) and artificial neural networks (ANN) towards efficient extraction of artemisinin from *Artemisia annua*. Ind. Crops Prod..

[17] Jiang H., Zhang Y., Cui R., Ren L., Zhang M., Wang Y. (2023). Effects of two different proportions of microbial formulations on microbial communities in kitchen waste composting. Microorganisms.

[18] Bagheri A.R., Ghaedi M., Dashtian K., Hajati S., Bazrafshan A.A. (2014). Simultaneous removal of Cu^2+^ and Cr^3+^ ions from aqueous solution based on complexation with eriochrome cyanine-R and derivative spectrophotometric method. Appl. Organomet. Chem..

[19] Baj T., Kowalska G., Kowalski R., Szymanska J., Kai G., Coutinho H.D.M., Sieniawska E. (2023). Synergistic antioxidant activity of four—Component mixture of essential oils: Basil, cedarwood, citronella and thyme for the use as medicinal and food ingredient. Antioxidants.

[20] Gasemloo S., Khosravi M., Sohrabi M.R., Dastmalchi S., Gharbani P. (2019). Response surface methodology (RSM) modeling to improve removal of Cr (VI) ions from tannery wastewater using sulfated carboxymethyl cellulose nanofilter. J. Clean. Prod..

[21] Wu J., Yao W., Zhao L., Zhao Y., Qi H., Zhang R., Song C., Wei Z. (2022). Estimating the synergistic formation of humus by abiotic and biotic pathways during composting. J. Clean. Prod..

[22] Xu J., Jiang Z., Li M., Li Q. (2019). A compost-derived thermophilic microbial consortium enhances the humification process and alters the microbial diversity during composting. J. Environ. Manag..

[23] Awasthi M.K., Selvam A., Lai K.M., Wong J.W.C. (2017). Critical evaluation of post-consumption food waste composting employing thermophilic bacterial consortium. Bioresour. Technol..

[24] (2016). National Food Safety Standards Determination of Fat in Food. https://img.antpedia.com/standard/files/pdfs_ora/20211002/GB%205009.6-2016.pdf.

[25] (2021). Organic Fertilizer. https://www.doc88.com/p-17939225808636.html.

[26] Ye Y., Xu J., Zhang Z., Zhang Y., Zhao Q., Xu J., Yuan H. (2024). Complex multi-dimensional integration for T2 and R2 mapping. Magn. Reson. Imaging.

[27] Filli K.B., Nkama I., Abubakar U.M., Jideani V.A. (2010). Influence of extrusion variables on some functional properties of extruded millet-soybean for the manufacture of *fura*: A Nigerian traditional food. Afr. J. Food Sci..

[28] Asadu C.O., Egbuna S.O., Chime T.O., Eze C.N., Kevin D., Mbah G.O., Ezema A.C. (2019). Survey on solid wastes management by composting: Optimization of key process parameters for biofertilizer synthesis from agro wastes using response surface methodology (RSM). Artif. Intell. Agric..

[29] Wang M., Liu Y., Wang S., Wang K., Zhang Y. (2021). Development of a compound microbial agent beneficial to the composting of Chinese medicinal herbal residues. Bioresour. Technol..

[30] Chen G., Chen J., Srinivasakannan C., Peng J. (2012). Application of response surface methodology for optimization of the synthesis of synthetic rutile from titania slag. Appl. Surf. Sci..

[31] Hidalgo D., Corona F., Martín-Marroquín J.M. (2022). Manure biostabilization by effective microorganisms as a way to improve its agronomic value. Biomass Convers. Bior..

[32] Xu J., Lu G., Shan G., He X., Huang J., Li Q. (2019). Inoculation with compost-born thermophilic complex microbial consortium induced organic matters degradation while reduced nitrogen loss during co-composting of dairy manure and sugarcane leaves. Waste. Biomass. Valori..

[33] Patil N., Mote G., Khot J.A., Prathapan K. (2025). Simplex lattice mixture design approach for optimization of jaggery granules and finger millet flour-based functional muffins. Sugar Tech..

[34] Khalid W., Koraqi H., Benmebarek I.E., Moreno A., Alsulami T., Mugabi R., Nayik G.A. (2025). Optimization of UAE-NADES green extraction of bioactive compounds from chickpea (*Cicer arietinum* L.) sprouts using simplex lattice mixture design methodology. Ultrason. Sonochem..

[35] Rodriguesa B., Hermana C. (2023). Optimization of the recovery yield of the enzymatic aqueous extraction of oil from wet açaí decocts using Design of Experiment. Grasas Aceites.

[36] Bezerra M.A., Lemos V.A., Novaes C.G., Mota de Jesus R., Filho H.R.S., Araújo S.A., Alves J.P.S. (2020). Application of mixture design in analytical chemistry. Microchem. J..

[37] Gou C., Wang Y., Zhang X., Lou Y., Gao Y. (2017). Inoculation with a psychrotrophic-thermophilic complex microbial agent accelerates onset and promotes maturity of dairy manure-rice straw composting under cold climate conditions. Bioresour. Technol..

[38] Zhang X., Song Y., Yang X., Hu C., Wang K. (2023). Regulation of soil enzyme activity and bacterial communities by food waste compost application during field tobacco cultivation cycle. Appl. Soil. Ecol..

[39] Hiaaham N.F.N., Kadir A.A., Sarani N.A., Hassan M.I.H., Fadeli M.F.A., Mazilan S.N. (2024). The evaluation of composted food waste effects in conserving and enhancing growth performance of *Azolla Pinnata*. Int. J. Conserv. Sci..

[40] Zhou X., Yang J., Xu S., Wang J., Zhou Q., Li Y., Tong X. (2020). Rapid in-situ composting of household food waste. Process Saf. Environ..

