# Supplementary material for: Optimization of Microbial Consortium Formulation for Oily Food Waste Composting Using Mixture Design Methodology
Source: Microorganisms. 2025 Sep 5;13(9):2066. doi: 10.3390/microorganisms13092066 (PMC12473027; doi:10.3390/microorganisms13092066)
Supplement: Supplementary file 1 [file microorganisms-13-02066-s001.zip › microorganisms-3749702-supplementary.pdf]

Optimization of microbial consortium formulation for oily food waste composting using mixture design methodology

Yun Zhang[1,2], Yujun Shen[1,2,*], Jingtao Ding[1,2], Haibin Zhou[1,2], Hang Zhao[1,2], Hongsheng Cheng[1,2], Pengxiang Xu[1,2], Yiwei Qin[1,2], Yang Jia[1,2]

[1] Institute of Energy and Environmental Protection, Academy of Agricultural Planning & Engineering, Ministry of Agriculture and Rural Affairs, Beijing 100125, China

[2] Key Laboratory of Technologies and Models for Cyclic Utilization from Agricultural Resources, Ministry of Agriculture and Rural Affairs, Beijing 100125, China

[*] Corresponding author, Tel: +86-010-59196810. Email Address: shenyujunaape@163.com

Contents:

Table S1 Programmed temperature rise settings

Figure S1 Effects of mixture bacteria on GI (a), pH (b), moisture content (c), C/N (d) during composting of food waste with cornstalk in the small-scale simulation fermenter

Figure S2 Effects of mixture fungi on GI (a), pH (b), moisture content (c), C/N (d) during composting of food waste with cornstalk in the small-scale simulation fermenter

Figure S3 Effects of mixture bacteria and fungi on GI (a), pH (b), moisture content (c), C/N (d) during composting of food waste with cornstalk in the small-scale simulation fermenter

Figure S4 The temperature changes during the two simulation experiments of the small-scale simulation fermenter (a) the first experiment, and (b) the second experiment

Table S2 Analysis of variance (ANOVA) for the bacterial, fungal and bacterial-fungal group

Table S1 Programmed temperature rise settings

Experiment number	The first experiment		The second experiment	
Stage	Temperature/℃	Operation time/d	Temperature/℃	Operation time/d
One	30	1	35	1
Two	40	1	45	1
Three	50	1	55	1
Four	55	1	65	8
Five	60	7	45	4
Six	40	4	50	4
Seven	45	4	35	9
Eight	30	8		

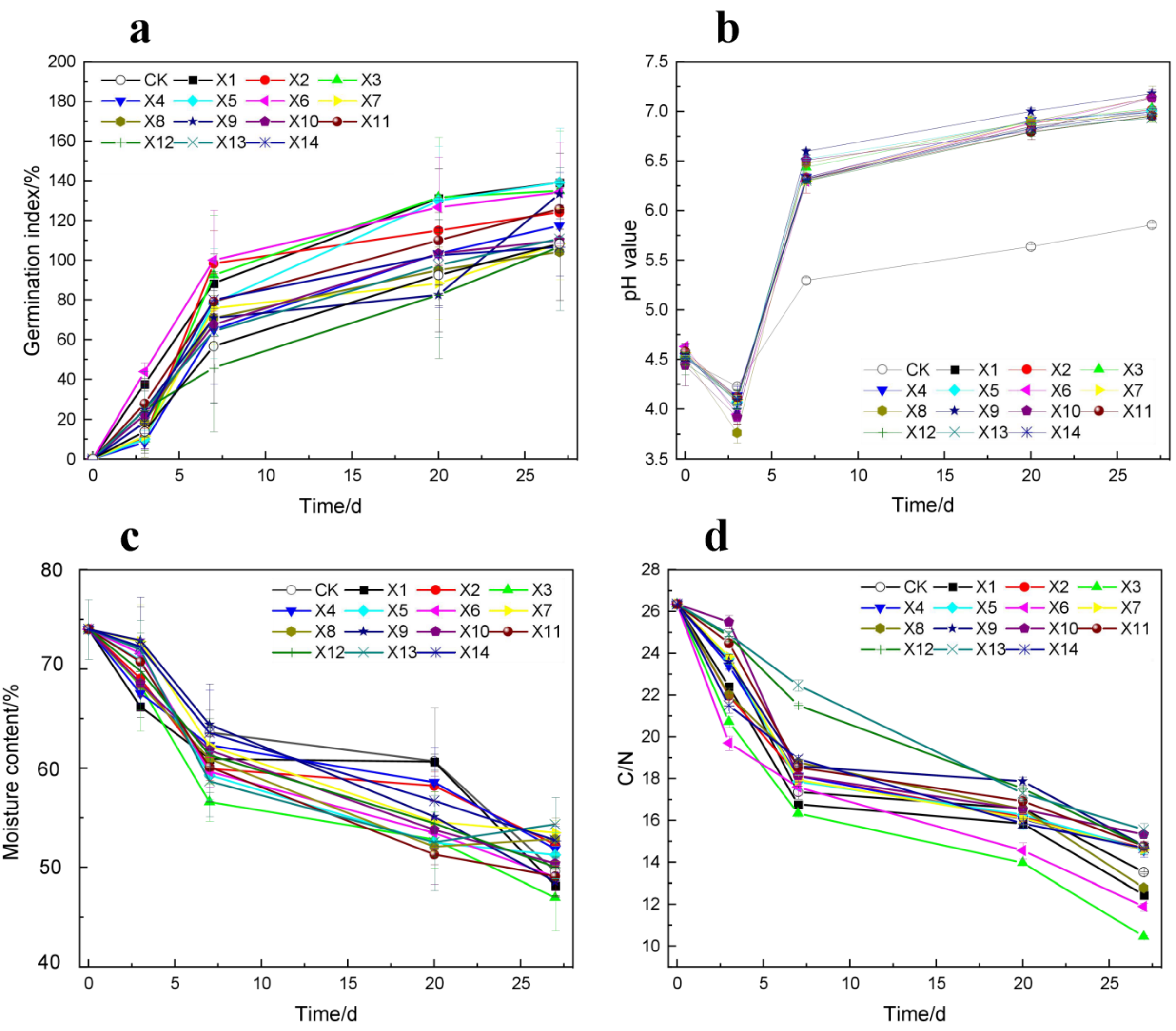

Figure S1 Effects of mixture bacteria on GI (a), pH (b), moisture content (c), C/N (d) during composting of food waste with cornstalk in the small-scale simulation fermenter (CK represents the blank control group without inoculation, while X1 to X14 denote bacterial treatments, with numbers 1 to 14 corresponding to the "Run" column in Figure 1)

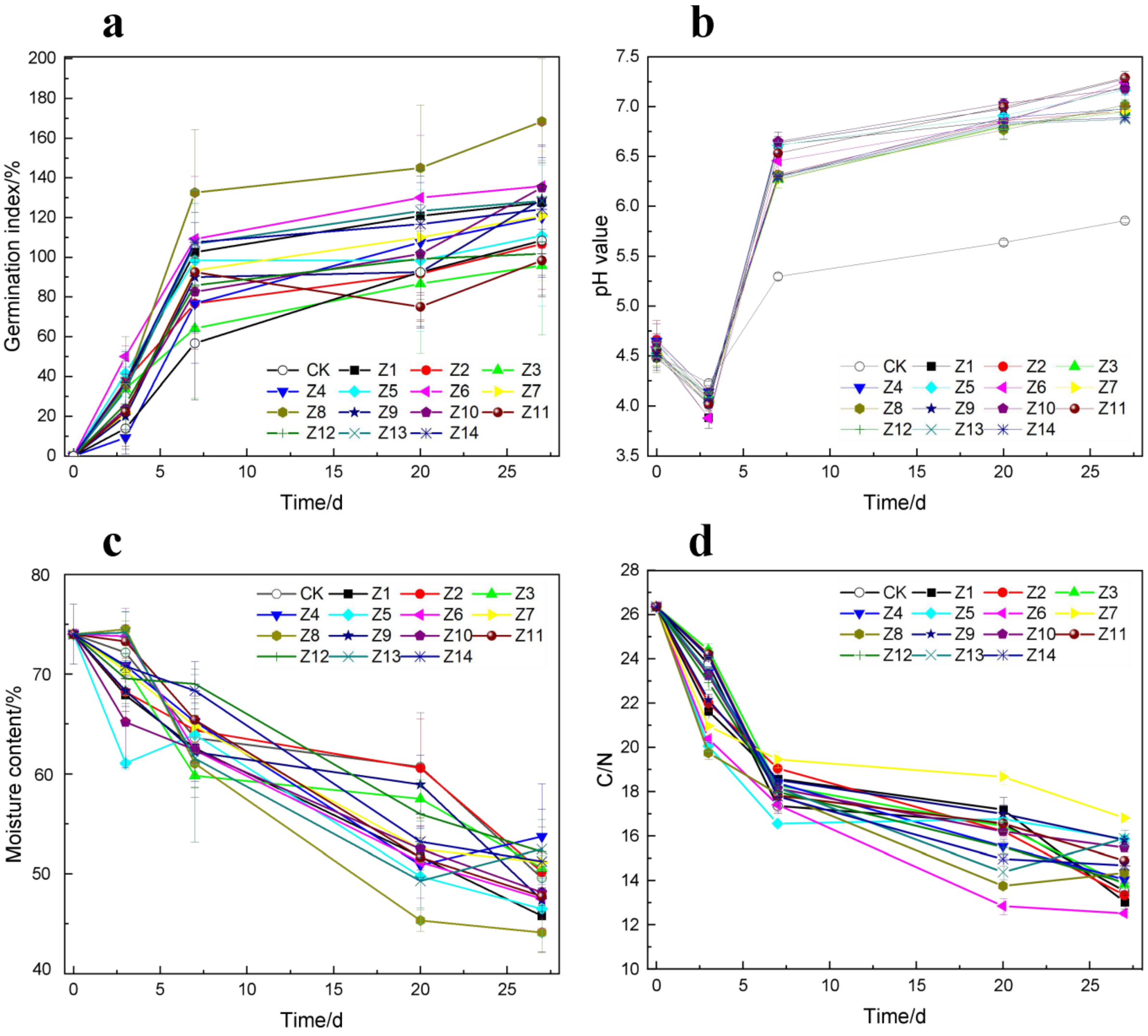

Figure S2 Effects of mixture fungi on GI (a), pH (b), moisture content (c), C/N (d) during composting of food waste with cornstalk in the small-scale simulation fermenter (CK represents the blank control group without

inoculation, while Z1 to Z14 denote fungal treatments, with numbers 1 to 14 corresponding to the "Run" column in Figure 1)

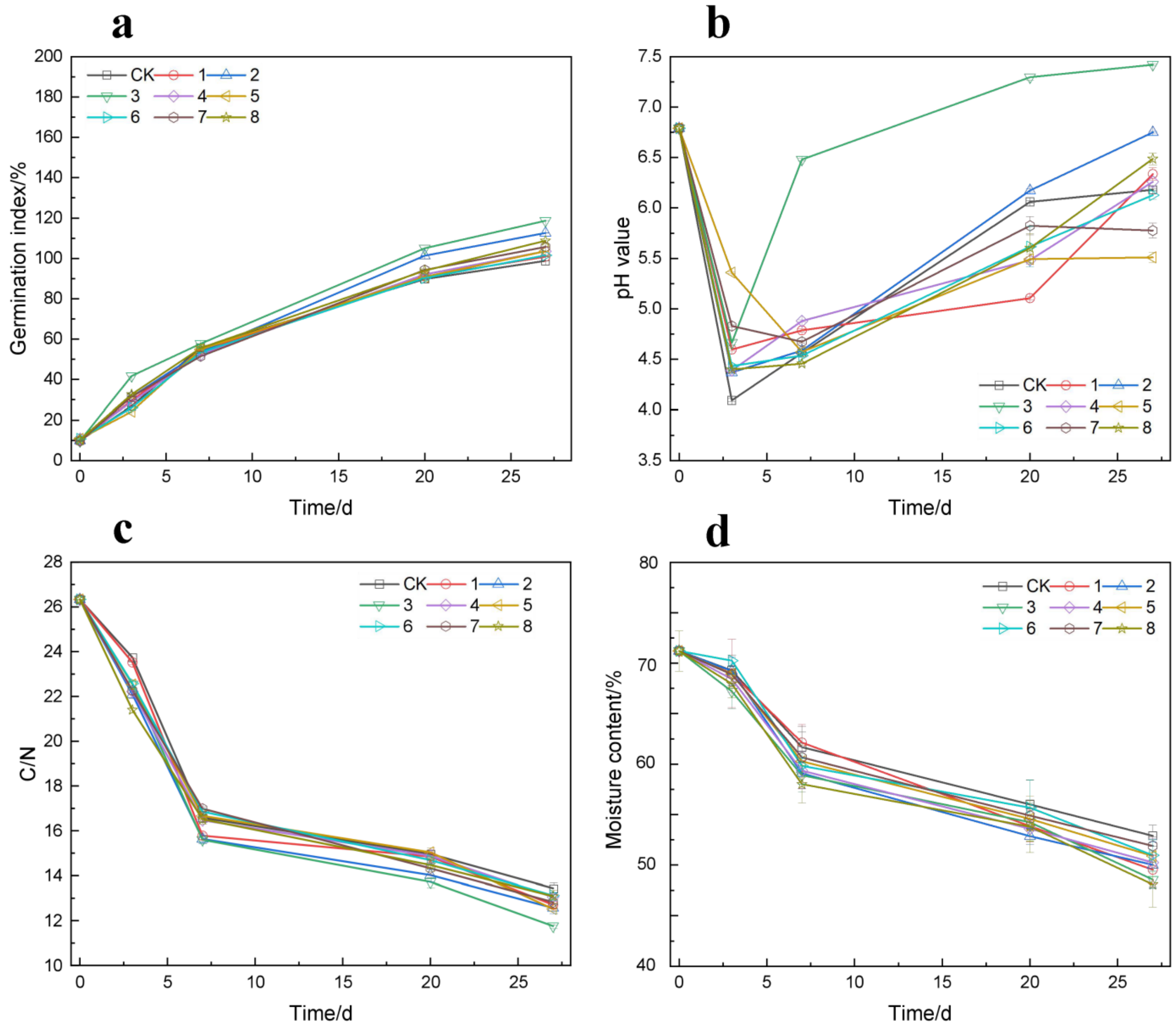

Figure S3 Effects of mixture bacteria and fungi on GI (a), pH (b), moisture content (c), C/N (d) during composting of food waste with cornstalk in the small-scale simulation fermenter (CK represents the blank control group without inoculation, while 1 to 8 denote bacterial-fungal treatments, with numbers 1 to 8 corresponding to the "Run" column in Figure 1)

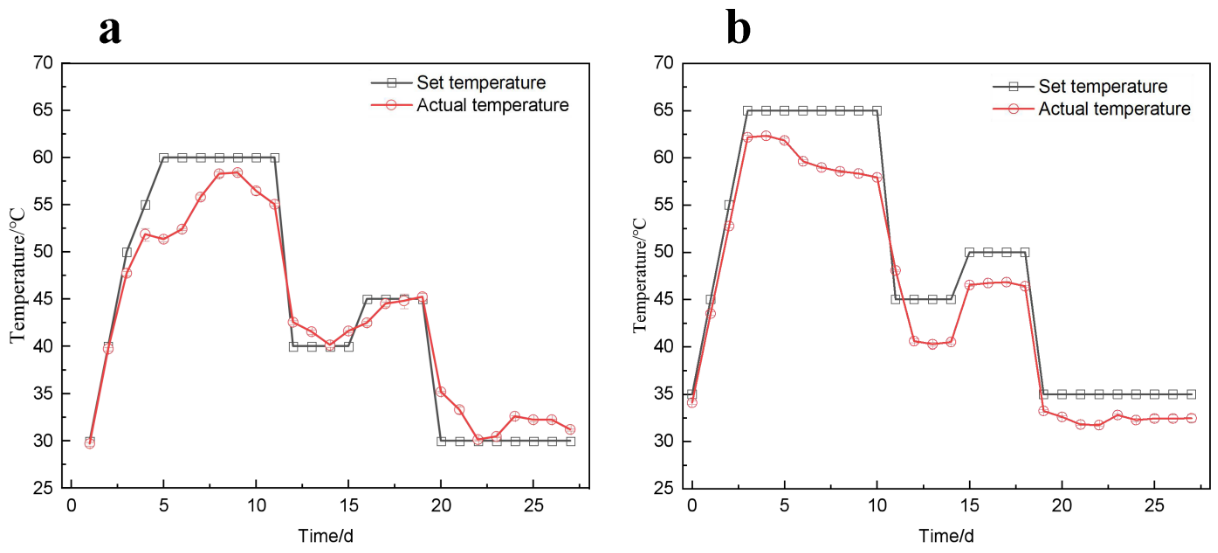

Figure S4 The temperature changes during the two simulation experiments of the small-scale simulation fermenter (a) the first experiment, and (b) the second experiment

Table S2 Analysis of variance (ANOVA) for the bacterial, fungal and bacterial-fungal group

Compounding group	Source	Sum of squares	df	Mean square	F-value	P-value	
	**Model**	567.18	6	94.53	28.45	0.0001	significant
	Linear Mixture	273.07	2	136.54	41.09	0.0001	
	AB	16.04	1	16.04	4.83	0.0640	
	AC	4.56	1	4.56	1.37	0.2800	
	BC	5.33	1	5.33	1.61	0.2457	
Bacterial	ABC	142.50	1	142.50	42.88	0.0003	
	**Residual**	23.26	7	3.32			
	Lack of Fit	4.22	3	1.41	0.2956	0.8278	not significant
	Pure Error	19.04	4	4.76			
	Cor Total	590.44	13				
	Std. Dev.	1.82				R^2	0.9606

	Mean	19.02				Adj. R^2	0.9268
	C.V. %	9.58				Pred. R^2	0.8258
						Adeq Precision	17.7901
Fungal	**Model**	810.26	6	135.04	40.25	< 0.0001	significant
	Linear Mixture	129.90	2	64.95	19.36	0.0014	
	AB	15.03	1	15.03	4.48	0.0721	
	AC	21.02	1	21.02	6.26	0.0408	
	BC	33.93	1	33.93	10.11	0.0155	
	ABC	217.64	1	217.64	64.86	< 0.0001	
	**Residual**	23.49	7	3.36			
	Lack of Fit	3.48	3	1.16	0.2317	0.8703	not significant
	Pure Error	20.01	4	5.00			
	Cor Total	833.75	13				
	Std. Dev.	1.83				R^2	0.9718
	Mean	26.34				Adj. R^2	0.9477
	C.V. %	6.96				Pred. R^2	0.8756
						Adeq Precision	20.3076
Bacterial-Fungal	**Model**	158.21	3	52.74	34.39	0.0026	significant
	Linear Mixture	27.11	1	27.11	17.68	0.0136	
	AB	113.74	1	113.74	74.17	0.0010	
	AB(A-B)	17.36	1	17.36	11.32	0.0282	
	**Residual**	6.13	4	1.53			
	Lack of Fit	2.18	1	2.18	1.66	0.2884	not significant
	Pure Error	3.95	3	1.32			
	Cor Total	164.34	7				
	Std. Dev.	1.24				R^2	0.9627
	Mean	13.17				Adj. R^2	0.9347
	C.V. %	9.40				Pred. R^2	0.8203
						Adeq Precision	13.8194
